# Association of rs1801157 single nucleotide polymorphism of CXCL12 gene in breast cancer in Pakistan and *in-silico* expression analysis of CXCL12–CXCR4 associated biological regulatory network

**DOI:** 10.7717/peerj.3822

**Published:** 2017-09-15

**Authors:** Samra Khalid, Rumeza Hanif

**Affiliations:** Atta-ur-Rahman School of Applied Biosciences (ASAB)/Assistant Professor/Healthcare Biotechnology, National University of Science and Technology, Islamabad, Pakistan

**Keywords:** CXCL12, SNP rs1801157, Breast cancer, PDZK1, PI3k/Akt

## Abstract

**Background:**

C-X-C chemokine ligand 12 (CXCL12) has important implications in breast cancer (BC) pathogenesis. It is selectively expressed on B and T lymphocytes and is involved in hematopoiesis, thymocyte trafficking, stem cell motility, neovascularization, and tumorigenesis. The single nucleotide polymorphism (SNP) rs1801157 of CXCL12 gene has been found to be associated with higher risk of BC.

**Methods:**

Our study focuses on the genotypic and allelic distribution of SNP (rs1801157; G/A) in Pakistani population as well as its association with the clinico-pathological features. The association between rs1801157 genotypes (G/A) and BC risks was assessed by a multivariate logistic regression (MLR) analysis. Genotyping was performed in both healthy individuals and patients of BC using PCR-restriction fragment length polymorphism (PCR-RFLP) method. Furthermore, *in-silico* approaches were adapted to investigate the association of CXCL12 and its receptor CXCR4 with genes/proteins involved in BC signalling.

**Results:**

Significant differences in allelic and genotypic distribution between BC patients and healthy individuals of genotype (G/G) and (A/G) (*p* < 0.05) were observed. The frequency of the allele G in the BC group (77%) was significantly higher as compared to control group (61%) (*p* = 0.01). The association of genotype GG with clinico-pathological features including age, stages of cancer and organ (lung, liver, bones and brain) metastasis (*p* > 0.05) was assessed. In a MLR analysis, a number of variables including age, weight of an individual, affected lymph nodes, hormonal status (estrogen and progesterone receptor), alcohol consumption and family history associated with the GG genotype (GG:AA, odds ratio (OR) = 1.30, 95% CI [1.06–1.60]) were found to be independent risk factors for BC. Our *in-vitro* results suggest that genotype GG is possibly increasing the risk of BC in Pakistani cohorts. *in-silico* analysis finds that CXCL12–CXCR4 is associated with an increased expression of PDZK1, PI3k and Akt which lead the breast tumor towards metastasis.

**Conclusion:**

Multiple targets such as CXCL12, CXCR4, PDZK1, PI3k and Akt can be inhibited in combined strategies to treat BC metastasis.

## Introduction

**Figure 1:**
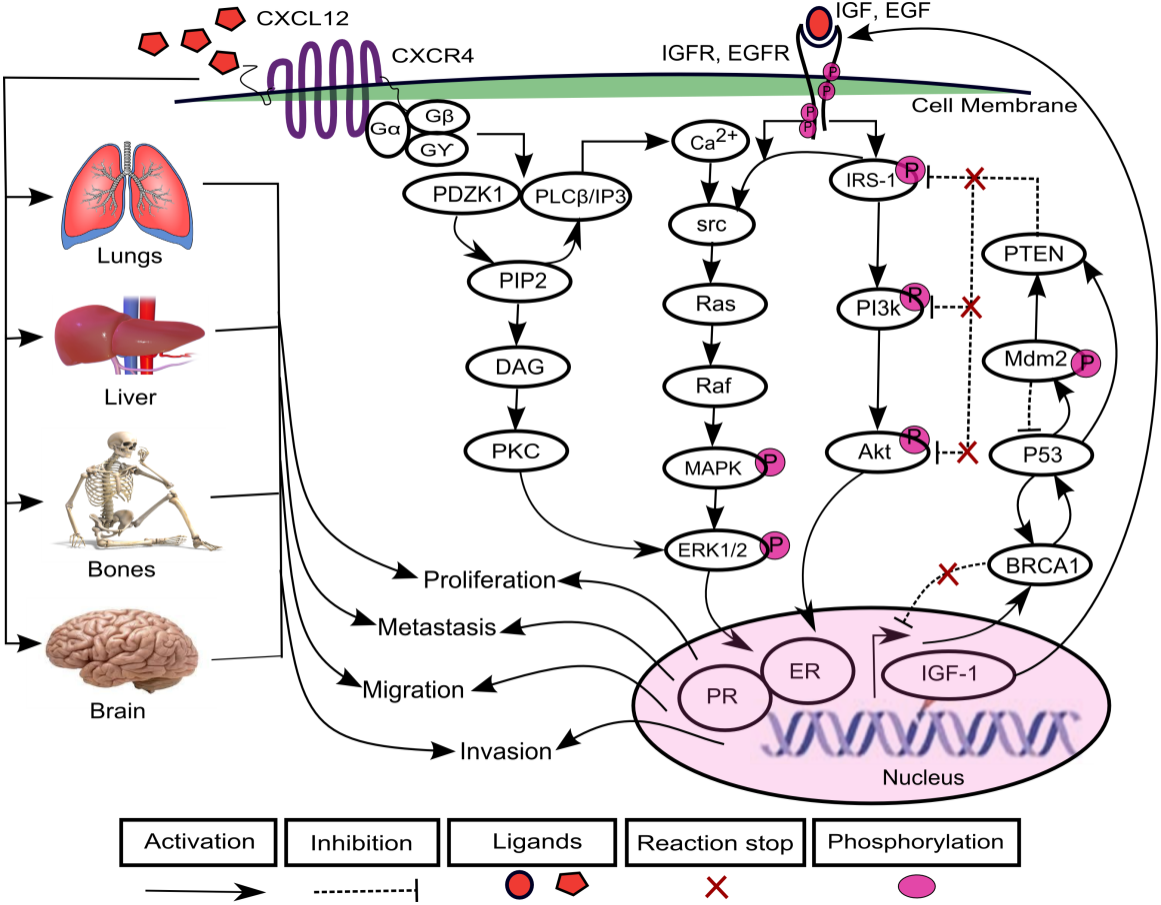
CXCL12–CXCR4 associated signaling pathways in BC. The binding of ligand CXCL12 with CXCR4, a G-protein coupled recepetor, is involved in the activation of signaling protein PDZ domain containing 1 (PDZK1). PDZK1 activates diacylglycerol (DAG) through hydrolyzes of phosphatidylinositol diphosphate (PIP2) to phosphatidylinositol triphosphate (IP3). DAG further induces activation of another mitogenic signaling pathway of extracellular signal regulated kinase1/2 (ERK1/2) through phospholipid dependent protein kinase C (PKC) that promotes cell proliferation. The direct interaction of PDZK1 with phospholipase C-β (PLC-β) promotes the calcium ions (Ca2+) signaling to activates proto-oncogenes src-Ras-Raf-mitogen activated protein (MAPK) kinases. It further enhances the signaling of growth promoting factors including estrogen growth factor EGF and insulin growth factor IGF that phosphorylate downstream mediator proteins including insulin receptor substrate-1 (IRS-1), phosphoinositide 3 kinase-serine/threonine protein kinases (PI3k-Akt) and MAPK-ERK1/2 kinases after binding with receptors EGFR and IGFR. CXCL12/CXCR4, EGF/EGFR and IGF/IGFR signaling can lead to increased expressions of estrogen and progesterone receptor (ER and PR) that lead the system towards proliferation, migration, invasion and metastasis. Furthermore, the cancer can metastasized to lungs, liver, bones and brain resulting from loss of function mutations of various tumor suppressor genes including phosphatase and tensin homolog (PTEN), BC susceptibility genes 1 (BRCA1), p53 and mouse double minute 2 homolog (Mdm2).

The increasing prevalence of breast cancer (BC) in Asia especially in Pakistan is a leading cause of cancer related mortalities in females (Asif et al., 2014). A positive correlation has been found between varying levels of chemokines and BC progression (Dewan et al., 2006; Gu et al., 2012; Hinton, Avraham & Avraham, 2010; Oliveira et al., 2011; Rhodes et al., 2011; Singh et al., 2013; Svensson et al., 2015; Tsuyada et al., 2012). Chemokines are cytokines that are involved in the regulation of leukocytes trafficking in both inflammatory and homeostatic states (Luster, 1998; Murdoch & Finn, 2000; Schiraldi et al., 2012). The chemokine (C-X-C motif) ligand 12 (CXCL12) is selectively expressed on B and T lymphocyte and is located on chromosome 10q11.1 (Baggiolini, Dewald & Moser, 1997; Shirozu et al., 1995; Tang et al., 2010; Watanabe et al., 2008). CXCL12 as a ligand interacts with chemokine (C-X-C motif) receptor 4 (CXCR4) and transduce signals involved in essential cellular processes such as cell cycle proliferation, survival, differentiation, apoptosis and cell chemotaxis (Dewan et al., 2006; Ganju et al., 1998; Luker & Luker, 2006; Qin et al., 2017; Schober et al., 2006; Wescott et al., 2016). Previous studies have shown an increased expression of *CXCL12* and *CXCR4* which allowed migration of cancer cells to metastatic organs such as lung, liver and bone and brain via a CXCL12–CXCR4 chemotactic gradient which leads towards metastasis (Cavallaro, 2013; Hinton, Avraham & Avraham, 2010; Liberman et al., 2012; Salomonnson et al., 2013; Sun et al., 2014). CXCL12 triggers dimerization of receptor CXCR4 which activates multiple signaling pathways including PDZ domain containing 1 (PDZK1), phosphoinositide 3 kinase-serine/threonine protein kinases (PI3k-Akt) (Teicher & Fricker, 2010) and mitogen activated kinase-extracellular signal regulated kinase1/2 (MAPK-ERK1/2) (Sobolik et al., 2014) (Fig. 1).

In one of the pathways, PDZK1 interacts with phospholipase C-β (PLC-β) which contributes to phosphorylation of ERK1/2 and calcium ion (Ca^2+^) channels in response to the activation of G-protein upon CXCL12 binding to CXCR4 (Kim et al., 2014; Neves, Ram & Iyengar, 2002). It further promotes hydrolysis of phosphatidylinositol diphosphate (PIP2) to diacylglycerol (DAG) and phosphatidylinositol triphosphate (IP3). DAG directly activates phospholipid dependent protein kinase C (PKC) involved in cell proliferation (Kim et al., 2014; Park et al., 2012). The calcium influx induces phosphorylation of proto-oncogene src (Xie et al., 2005) which activates their associated effector proteins Ras-Raf and PI3k-Akt through phosphorylated insulin receptor substrate-1 (IRS-1) (Irby & Yeatman, 2000; Pruett et al., 1995; Roberts & Der, 2007). The overexpression of PDZK1, a newly identified estrogen regulator protein, enhances activity of growth promoting factors including estrogen growth factor receptor (EGFR) and insulin-like growth factor receptor (IGF-1R) and their related signaling pathways associated with BC (Kim et al., 2014). It can lead to an increased expression of estrogen and progesterone receptor (ER and PR) that are translocated in the nucleus, causing loss of function mutations of tumor suppressor genes (TSGs) such as phosphatase and tensin homolog (PTEN), BC susceptibility genes 1 (BRCA1), p53 and mouse double minute 2 homolog (Mdm2) (Bailey et al., 2012; Daniel, Hagan & Lange, 2011; Khalid et al., 2016; Liu et al., 2006; Werner & Maor, 2006).

Single nucleotide polymorphism (SNP) rs1801157 also recognized as G801A is located on exon 4 of β splice variant in CXCL12 gene transcripts (Hirata et al., 2007). This SNP involves a guanine to adenine (G → A) substitution at base pair 801 of the 3′-untranslated region of CXCL12 gene (Modi et al., 2005; Petersen et al., 2005) which is associated with BC progression in various studies (Chang et al., 2009; Chen et al., 2006; Hidalgo-Pascual et al., 2007; Maley et al., 2009). Due to a known metastatic function of chemokine, CXCL12 variant is considered as a risk factor and has been previously reported in association with multiple cancers including myeloma (Luker & Luker, 2006; Pemberton et al., 2006), colorectal (Hidalgo-Pascual et al., 2007), cervical (Maley et al., 2009), basal cell (Chen et al., 2006) and breast carcinoma (Luker & Luker, 2006). The allelic and genotypic frequencies of SNP rs1801157 of CXCL12 gene are studied to enhance the interaction with CXCR4. This known interaction has been shown to increase pathogenesis and disease susceptibility of multiple diseases (Ma et al., 2012).

In BC, the expression analysis of CXCL12 gene and its association with tumor stages has been evaluated in different inhabitants all over the world (De Oliveira et al., 2009; Kato et al., 2003; Oliveira et al., 2011; Schimanski et al., 2011). The allele A was associated to be a risk factor among BC patients especially in Asian ethnicity (De Oliveira et al., 2009). So far this association of SNP rs1801157 of CXCL12 gene has not been investigated in Pakistani population suffering from BC. This study aims to report the incidence of SNP (rs1801157; G/A) of CXCL12 gene and its association with clinico-pathological features and risk factors of BC in Pakistani population. In addition, the changes in expression level of genes and proteins involved in CXCL12–CXCR4 associated Biological Regulatory Network (BRN) were analyzed by *in-silico* experiments. The generalized computational modeling of Rene’ Thomas (Thomas, 1973; Thomas, 2002; Thomas & Kaufman, 2001a; Thomas & Kaufman, 2001b; Thomas & Kaufman, 2002; Thomas, Thieffry & Kaufman, 1995) provide a dynamical insights to study the molecular mechanism of involvement of CXCR4 and CXCL12 in the metastasis of BC.

## Materials and Methods

The present study is based on both *in-vitro* and *in-silico* experiments.

### Blood sample collection

This study was approved by the ethics committee of the Department of Atta-ur-Rahman School of Applied Biosciences, National University of Science and Technology (NUST) and the hospitals from where the blood samples were collected. A written consent was signed prior to blood sampling from all donors. Samples were provided by the NORI hospital Islamabad and Holy Family hospital, Rawalpindi in Pakistan. Peripheral blood from 218 BC patients and 147 non-BC individuals were collected in sterile vacutainers (BD Vacutainer^®^, Franklin Lakes, NJ, USA) having K3 ethylene diamine tetra acetate (EDTA). Samples were stored in 4 °C prior to DNA extraction.

### DNA isolation

Genomic DNA isolation protocol was performed as proposed by Sambrook, Fritsch & Maniatis (1989). Phenol-chloroform extraction method was used to lyse blood cells which were de-proteinized with solvents such as phenol, chloroform and isoamyl alcohol (Sigma-Aldrich, St. Louis, MO, USA). DNA pellet was re-suspended with proteinase K (Invitrogen, Carlsbad, CA, USA) (25 µg/mL) solution for the separation of polypeptides. 0.2 ml TE buffer (10 mM Tris-HCl, 1 mM EDTA, pH 8.0) was added in isolated genomic DNA and was kept overnight after washing with 70% ethanol (Sigma-Aldrich, St. Louis, MO, USA) and was stored at 4 °C until use. Concentration of DNA was measured using spectrophotometer (SP300 Optima; Optima Inc., Tokyo, Japan).

### PCR restriction fragment length polymorphism

An SNP (rs1801157; G/A) of CXCL12 genotyping was analyzed with specific primers reported in paper (Oliveira et al., 2011). A total of 300 ng of genomic DNA was amplified in a total PCR reaction mixture of 25 µl containing 1.25U Taq DNA polymerase (Fermentas; Thermo Scientific, Waltham, MA, USA). Genotyping was done using PCR restriction fragment length polymorphism (PCR-RFLP). DNA was amplified at 94 °C for 5 min, followed by 35 cycles of 30 s at 94 °C, 30 s at 56 °C for primers to hybridize and 40 s at 72 °C followed by 7 min extension step at 72 °C in a thermal cycler (Applied Biosystems, Foster city, CA, USA). The PCR fragment run on 2% agarose gel was of 293 bp size.

### CXCL12 rs1801157 genotyping

PCR products were assimilated with 10 U/µl concentration of the MspI (Fermentas, Waltham, MA, USA) enzyme at room temperature for 2 h. The bands of restriction digestion of genotype GG, AG and AA were visualized using UV transilluminator (Dolphin Doc; Wealtham, New York, NY, USA). Assimilation of G/G genotype produces two fragments of 193 and 100 bp while the AA genotype yielded only 293 bp product.

### Statistical analysis

Statistical analysis of allelic frequencies of SNP (rs1801157; G/A) of CXCL12 gene in both BC and non-BC groups was calculated by Hardy Weinberg equilibrium ([1 × (h + 2H)]/2N, where “h” represents as heterozygous genotype, “H” for homozygous and “N” represents the number of samples. A chi-square (χ2) test of both genotypic and allelic frequencies were considered as statistically significant (*p* < 0.05) using GraphPad PRISM statistic software (version 7.0) (GraphPad Software, La Jolla, CA, USA). Associations between the clinic-pathological features (age, stages and organ metastasis) and the presence of BC were evaluated using One-Way ANOVA (Bonferroni test). The effects of variables including age (equal to or below or above 60), overweight, lymph node status, hormonal status (estrogen and progesterone receptor), alcohol and family history associated with rs1801157 genotypes was calculated as reported previously by using multivariate logistic regression (MLR) analysis (Wang et al., 2003).

### *In-silico* analysis of CXCL12–CXCR4 associated Biological Regulatory Network (BRN) using Kinetic Logic Formalism

The discrete modeling of Biological Regulatory Network (BRN) was first introduced by Thomas (1973). The kinetic logic formalism was used to analyze the behaviours of genes and proteins involved in BRN. The dynamics of Rene’ Thomas formalism has been provided from Ahmad et al. (2007), Ahmad et al. (2012) and Khalid et al. (2016). These dynamics are specified as respective differential equation of the system. The constructed BRN model was then applied to the software, GENOTECH, to perceive the suitable logical parameters to produce an asynchronous state graph (Bernot et al., 2004). A BRN consisted of two main types of biological regulations which were activation and inhibition of node (representing genes/proteins) that represent the up and down regulation of a specific protein. In a dynamical network, nodes are connected with edges (represent as directed arrows) which are used to identify the behaviours of complex dynamical interactions among genes and environmental changes involved in signaling network.

## Results

**Figure 2:**
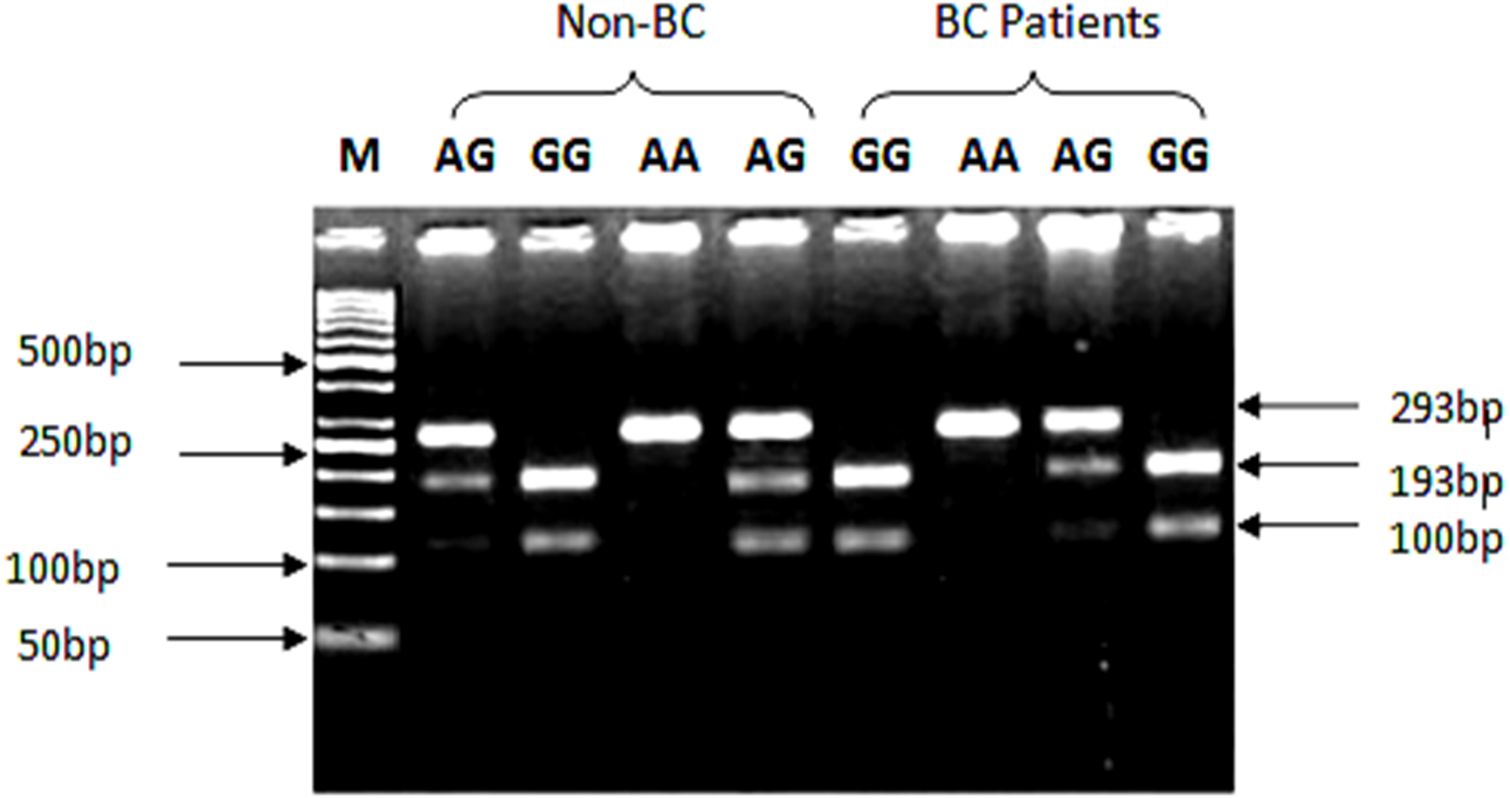
Genotypic variations of SNP (rs1801157; G/A) of CXCL12 gene in cases and control groups. The gDNA samples were run on 2% agarose gel in the following order from left to right: lane 1: 50 bp marker, lanes 2 & 5 contain heterozygous AG genotype, lane 3 contains homozygous GG genotype while lane 4 contains AA genotype from non-BC individuals. Lanes 6 & 9 contain homozygous GG genotype, lane 7 contains AA genotype, while lane 8 contains heterozygous AG genotype from BC patients.

Genotyping of SNP rs1801157 of CXCL12 gene was analyzed through PCR-RFLP. The PCR products were digested with MspI enzyme (Fermentas, Waltham, MA, USA) capable of recognizing sequence 5′-CCGG-3′ in allele G. After amplification of CXCL12 gene with Msp1 digestion, allele G produced two fragments of 100 bp and 193 bp product lengths, while the AA genotype yielded a 293 bp product shown in Fig. 2.

### Analysis of Genotypic (GG, AG and AA) and Allelic (G/A) distribution

**Figure 3:**
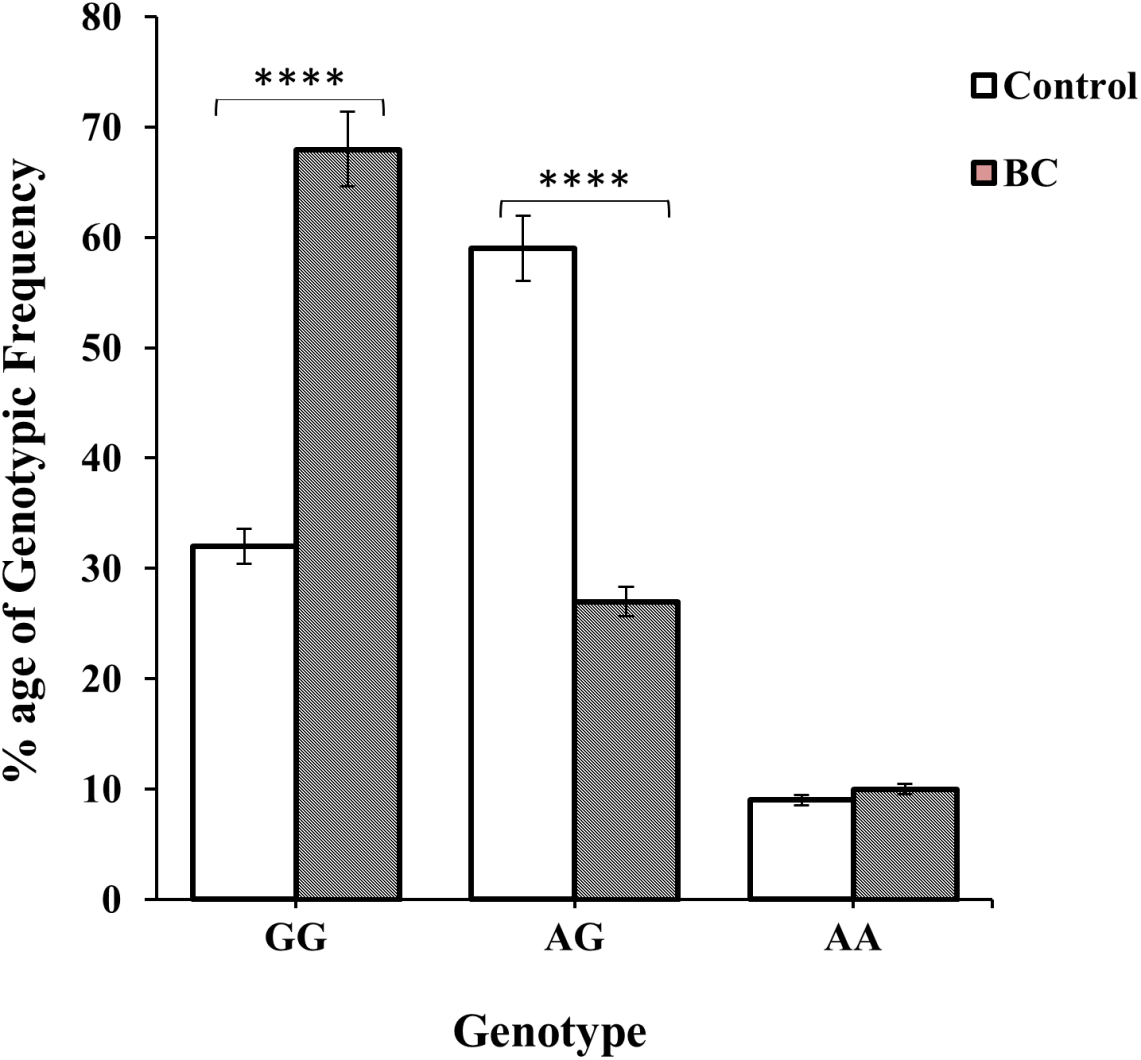
Genotypic frequency of CXCL12 rs1801157 in the control group and BC patients. The difference of genotype frequencies among patients and healthy subjects, *χ*2 = 38.85 (two degrees of freedom; *p* < 0.01), is statistically significant.

Genotype GG and AG were more prevalent among BC and non-BC individuals i.e., 138 (63%) BC patients and 86 (59%) controls were observed as shown in Table 1 and Fig. 3. The frequency of homozygous AA genotype was 21 (10.0%) in cases and 14 (9.0%) in controls; while the frequency of heterozygous AG genotype was 59 (27.0%) and 86 (59.0%) in cases and controls, respectively. The frequency of homozygous GG genotype was significantly higher in BC cases 138 (63.0%) as compare to controls 47 (32.0%) (χ2 = 38.85:d2; *p* < 0.0001) (Table 1 and Fig. 3). Similarly, the frequency of allele G was found significantly higher in BC patients (OR 0.467 (0.25–0.86); *P* = 0.01) (Table 1).

**Table 1:** Genotypes and Alleles frequencies in cases and controls.

Genotypic frequency^a^				Allelic frequency^b^				OR (95% CI)^c^
Genotypes	Non-BC-control (*n* = 147)	BC Patients (*n* = 218)	*P* value (chi-square (*χ*2))	Alleles	Non-BC-control (*n* = 147)	BC Patients (*n* = 218)	*P* Value (chi-square ((*χ*2))	
GG	47 (32%)	138 (63%)	<0.0001*** (**38.85**)	G (%)	61	77	0.01* (**5.984**)	0.467 (0.25–0.86)
AG	86 (59%)	59 (27%)		A (%)	39	23		
AA	14 (9%)	21 (10%)						

**Notes.**

a BC patients × non-BC-control group 1; (****χ2 = 38.85:d2; *p* < 0.01).

b BC patients × non-BC-control group 1; *χ2 in HWE = 5.984 (one degree of freedom (d1); *p* < 0.05).

c OR, Odds Ration; CI, Confidence interval.

The genotypic frequencies of co-dominant model of genotype A/G was found to be significant (*p* < 0.01; OR: 3.79; 95% CI [2.44–5.92]) among non-BC individuals while dominant G/G genotype were more prevalent in BC patients i.e., *p* < 0.01; OR: 0.27; 95% CI [0.17–0.42] (Table 2 and Fig. 3). Whereas non-significant association (*p* = 0.97; OR: 0.98; 95% CI [0.48–2.01]) were of recessive AA genotype. It was observed that genotypic frequency of GG genotype was higher 138 (63%) in BC patients than the control group 47 (32%). A large number 59% of non-BC individuals of genotype AG was found to be significant than the BC patients (59) 27% shown in Table 2.

**Table 2:** Genotypic frequencies of SNP rs1801157 of CXCL12 gene according to dominant, co-dominant and recessive models in BC patients and control group.

Genotypic frequency				
Genotypes	Non-BC-control (*n* = 147)	BC Patients (*n* = 218)	*P* Value (χ2) (one-tailed)	OR (95% CI)^a^
Co-dominant model				
AG *n* (%)	86 (59%)	59 (27%)	<0.0001^****^ (**36.24**)	3.79 (2.44–5.92)
GG + AA *n* (%)	61 (41%)	159 (73%)		
Dominant model				
GG *n* (%)	47 (32%)	138 (63%)	<0.0001^****^ (**34.48**)	0.27 (0.17–0.42)
AG + AA *n* (%)	100 (68%)	80 (37%)		
Recessive model				
AA *n* (%)	14 (9%)	21 (10%)	0.97 (0.001)	0.98 (0.48–2.01)
AG + GG *n* (%)	133 (91%)	197 (90%)		

**Notes.**

**** χ2 = 36.24 co-dominant (A/G) and 34.48 dominant (G/G) genotype, d1; *p* < 0.01.

χ2 = 0.001 recessive genotype (A/A), d1; *p* > 0.05.

a OR, Odds Ration; CI, Confidence interval.

### Analysis of genotypic frequencies of CXCL12 associated SNP (rs1801157; G/A) with clinico-pathological features and factors in BC patients

**Figure 4:**
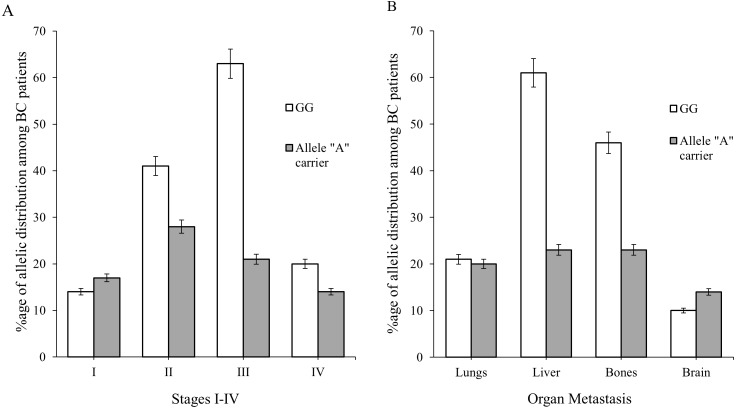
Allelic distribution of SNP rs1801157 of CXCL12 gene in BC patients was observed graphically. (A) The difference of allelic frequencies between patients of advanced stages I, II, III & IV were three degrees of freedom; *p* > 0.05. (B) The difference among organ metastasis in BC patients were three degrees of freedom; *p* > 0.05.

BC patients (of ages 20–60) in combination with clinico-pathologial features such as I–IV stages of BC and organ metastasis (lungs, liver, bones and brain) were used to find the association of genotype “GG” with allele “A” carrier. The association of genotype GG were found to be 46 (33%), 67 (49%) and 25 (18%) verses allele A carrier 30 (37%), 41 (51%) and 9 (11%) in age group between 20 and >60 of the patients (Table 3). Majority of patients were diagnosed with stage II (69, 32%) & III (84, 39%) respectively. While a small number of BC patients had stage I & IV i.e., 31 (14%) and 34 (15%), respectively as shown in Table 3 and Fig. 4A. The genotypic frequencies of genotype GG were found to be associated with large number of liver (61, 44%) and bone (46, 33%) metastasis verses allele A carrier (23, 29%) (Table 3 and Fig. 4B). While a small number of patients was diagnosed with lung (21, 15%) and brain (10, 7%) metastasis respectively shown in Table 3 and Fig. 4B.

**Table 3:** SNP (rs1801157; G/A) of CXCL12 gene associated with clinico-pathological features (age, stages and organ metastasis) of BC patients (*n* = 218).

		Total *N* (%) (*n* = 218)	**Genotype**		*p* value
			GG *N* (%)	Allele A carrier *N* (%)	
Age (years)	20–45	76 (35%)	46 (33%)	30 (37%)	0.1654
	46–60	108 (50%)	67 (49%)	41 (51%)	
	>60	34 (15%)	25 (18 %)	9 (11%)	
Tumor stage	I	31 (14%)	14 (10%)	17 (21%)	0.369
	II	69 (32%)	41 (30%)	28 (35%)	
	III	84 (39%)	63 (46%)	21 (26%)	
	IV	34 (15%)	20 (14%)	14 (18%)	
Tumor organ histology	Lungs	41 (19%)	21 (15%)	20 (25%)	0.349
	Liver	84 (38%)	61 (44%)	23 (29%)	
	Bones	69 (32%)	46 (33%)	23 (29%)	
	Brain	24 (11%)	10 (7%)	14 (18%)	

A multivariable logistic regression analysis with the various rs1801157 genotypes was conducted to evaluate the effect of seven factors where G/G compared with A/A genotype, age (equal to or below or above 60), weight of an individuals, affected lymph nodes, hormonal status (estrogen and progesterone receptor), alcohol consumption and family history remained as independent risk factors for BC (see all of ORs and 95% CIs in Table 4).

**Table 4:** Factors associated with the presence of BC in the multivariate analysis.

Factors	Category	*P* value	OR	95% CI
SNP rs1801157 genotypes	G/G	0.014	**1.30**	**1.06–1.60**
	A/A		1.00	
Age	Equal to or below or above 60	0.614	**0.93**	**0.70–1.24**
Overweight	Present	0.03	**1.29**	**1.03–1.63**
Lymph node status	Metastasis present	0.005	**1.24**	**1.07–1.44**
Hormonal status	ER	0.027	**1.20**	**1.02–1.40**
	PR	0.023	**1.30**	**1.04–1.63**
Alcohol	Present	0.84	0.96	0.64–1.43
Family history	Present	0.037	**1.95**	**1.04–3.66**

**Notes.**

Abbreviations ER estrogen receptor PR progesterone receptor OR odds ration CI confidence interval

### Construction of CXCL12–CXCR4 associated Biological Regulatory Network (BRN)

**Figure 5:**
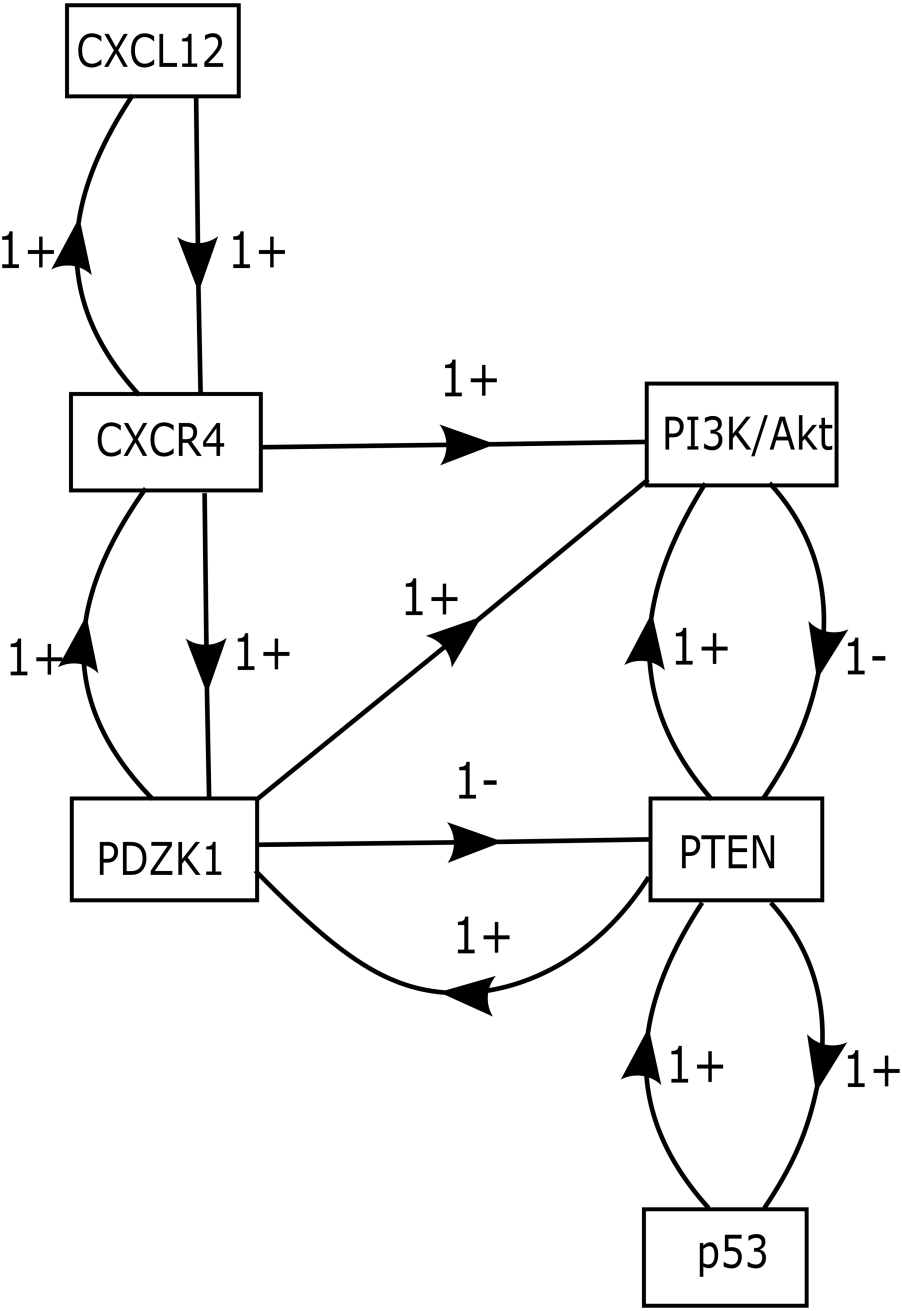
CXCL12–CXCR4 associated BRN model. Directed arrows represent activation (+ sign) and inhibition (− sign) among entities involved in the BRN model. The BRN has set of 6 biological entities *N* = {CXCL12, CXCR4, PDZK1, PTEN, PI3k/Akt and p53} directed as {(CXCL12 → CXCR4), (CXCR4 → PI3k/Akt), (CXCR4 → PDZK1), (PDZK1 → PI3k/Akt), (PDZK1 → PTEN), (PTEN → PI3k/Akt), (PI3k/Akt → PTEN), (PTEN → p53), (p53 → PTEN), (PTEN → PDZK1), (PDZK1 → CXCR4), (CXCR4 → CXCL12)}.

The role of CXCR12–CXCR4 in regulating estrogen and progesterone receptor (ER and PR) involved in BC signaling pathway are shown in Fig. 1. The model of CXCL12–CXCR4 associated BRN was constructed (shown in Fig. 5) to observe the complex dynamical interactions and behaviours of entities such as PDZK1, PI3k/Akt, PTEN and p53. The formal method of BRN modeling is a traditional approach which permits us to define the complexity of biological system which was more complex to identify through *in-vitro* experiments. A BRN consisted of two main types of biological regulations are activation (represent as positive sign) and inhibition (represent as negative sign) that have been achieved through previous experimental findings (Khalid et al., 2016; Kim et al., 2014; Sobolik et al., 2014; Teicher & Fricker, 2010). Experimental data was used to further validate the expression levels of each entity which interlinked at diverse points, related to CXCL12–CXCR4 associated BRN. We selected the key entities in our BRN to determine the significance of PDZK1, PI3k/Akt and tumor suppressor genes (PTEN and p53) in relation with overexpression of CXC12/CXCR4.

### Isolation of logical parameters of proteins involved in BRN

Our BRN model has six biological entities: CXCL12, CXCR4, PDZK1, PTEN, PI3k/Akt and p53 (Fig. 5). These six entities have set of logical parameters which govern the behaviour of each entity related to CXCL12–CXCR4 associated BRN model (Table 5). Its parametric values represent the level of each property which has closer approximation to biological system. CXCL12 physically interact with CXCR4 wherein PDZK1 must be present to activate PI3k/Akt signaling (given by parameter *K*_(PDZK1,{CXCR4,PI3k∕Akt})_ = 1). Active PDZK1 also leads to the suppression of PTEN which disrupts normal function of p53 (given by parameters *K*_(PDZK1,{PTEN})_ = 0, *K*_(PTEN,{p53})_ = 0, *K*_(PTEN,{PI3k∕Akt,p53})_ = 0).

**Table 5:** Logical parameters of each entity/protein involved in BRN model. The entities in braces represent the set of resources available for the respective protein and the numbers 0 and 1 represent the level of each property in BRN.

S.No.	Proteins	Logical parameters
1	CXCL12	*K*_(CXCL12,{})_ = 0, *K*_(CXCL12,{CXCR4})_ = 1
2	CXCR4	*K*_(CXCR,{})_ = 0, *K*_(CXCR4,{PDZK1})_ = 1, *K*_(CXCR4,{PI3k∕Akt})_ = 1, *K*_(CXCR4,{CXCL12})_ = 1, *K*_(CXCR4,{CXCL12,PI3k∕Akt})_ = 1, *K*_(CXCR4,{CXCL12,PDZK1})_ = 1, *K*_(CXCR4,{PDZK1,PI3k∕Akt})_ = 1, *K*_(CXCR4,{CXCL12,PDZK1,PI3k∕Akt})_ = 1
3	PDZK1	*K*_(PDZK1,{})_ = 0, *K*_(PDZK1,{PI3k∕Akt})_ = 1, *K*_(PDZK1,{PTEN})_ = 0, *K*_(PDZK1,{CXCR4})_ = 1, *K*_(PDZK1,{PTEN,PI3k∕Akt})_ = 0, *K*_(PDZK1,{CXCR4,PI3k∕Akt})_ = 1, *K*_(PDZK1,{CXCR4,PTEN})_ = 0, *K*_(PDZK1,{CXCR4,PTEN,PI3k∕Akt})_ = 1
4	PTEN	*K*_(PTEN,{})_ = 0, *K*_(PTEN,{p53})_ = 0, *K*_(PTEN,{PI3k∕Akt})_ = 1, *K*_(PTEN,{PDZK1})_ = 1, *K*_(PTEN,{PI3k∕Akt,p53})_ = 0, *K*_(PTEN,{PDZK1,p53})_ = 0, *K*_(PTEN,{PDZK1,PI3k∕Akt})_ = 1, *K*_(PTEN,{PDZK1,PI3k∕Akt,p53})_ = 0
5	PI3k/Akt	*K*_(PI3k∕Akt,{})_ = 0, *K*_(PI3k∕Akt,{PDZK1})_ = 1, *K*_(PI3k∕Akt,{PTEN})_ = 0, *K*_(PI3k∕Akt,{CXCR4})_ = 1, *K*_(PI3k∕Akt,{CXCR4,PDZK1})_ = 1, *K*_(PI3k∕Akt,{PDZK1,PTEN})_ = 0, *K*_(PI3k∕Akt,{CXCR4,PTEN})_ = 0, *K*_(PI3k∕Akt,{CXCR4,PDZK1,PTEN})_ = 1
6	p53	*K*_(p53,{})_ = 0, *K*_(p53,{PTEN})_ = 1

#### Analysis of CXCL12 associated BRN

**Figure 6:**
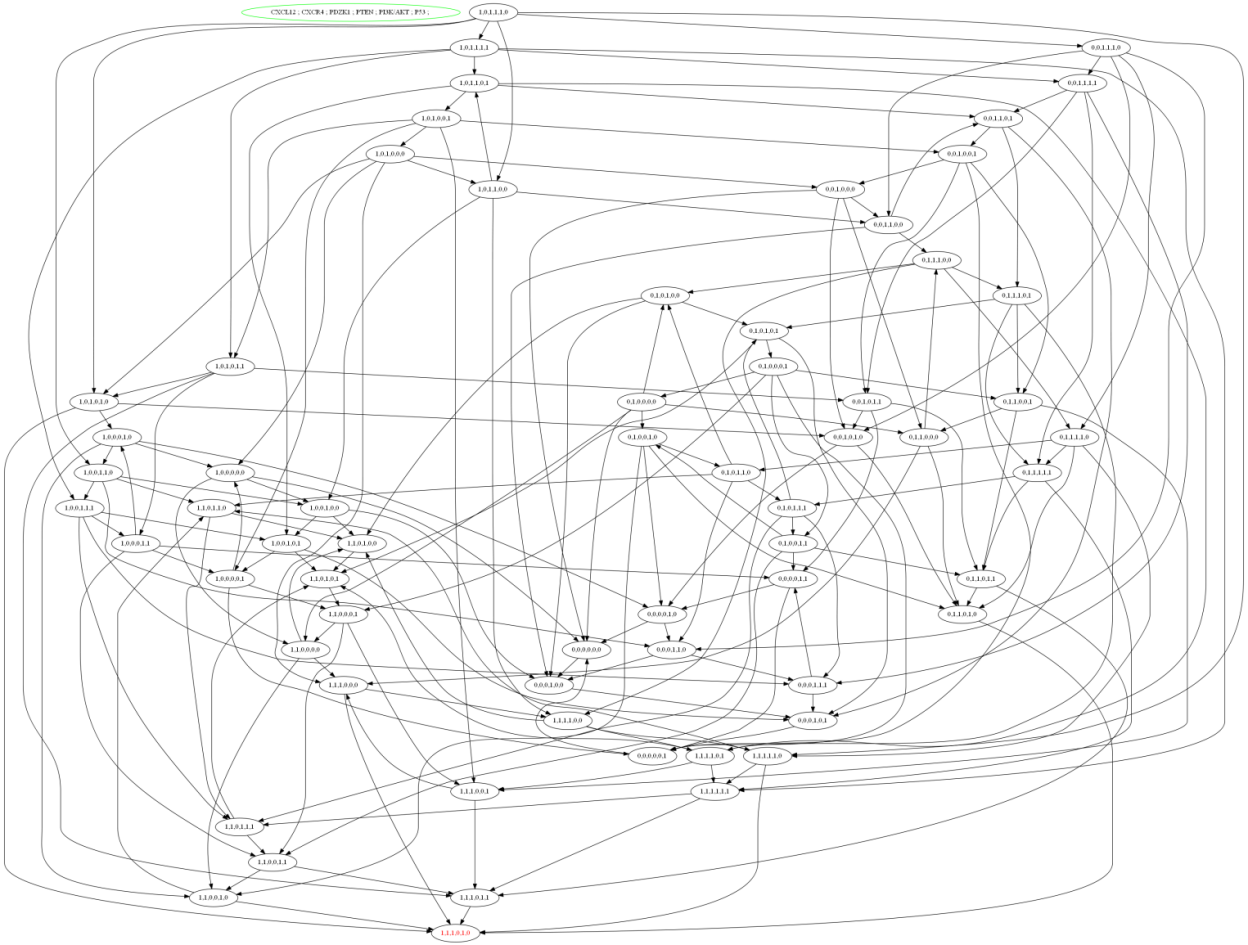
State graph of CXCL12 associated BRN. The state graph is generated by the set of logical parameters in BRN using the tool GENOTECH. In state graph, circles are represented as states where all the cyclic trajectories (directed arrows) are connected allowing passages from one state to another except deadlock state. The deadlock state (1, 1, 1, 0, 1, 0) represent the levels of CXCL12, CXCR4, PDZK1 and PI3k/Akt remain as active at metastatic level while the TSGs (PTEN and p53) are suppressed that lead the system towards BC progression.

The selected logical parameters of CXCL12–CXCR4 associated BRN were then applied to software, GENOTECH (version 3.0), to generate an asynchronous graph with initial and metastatic deadlock state (1, 1, 1, 0, 1, 0) shown in Fig. 6. The state graph contained 64 states and 51 cyclic trajectories (representing as directed arrows among states) (see Table S1) to show the relative expression levels of entities/proteins with respect to each other’s biological behaviour. These unique cyclic trajectories represent how 51 cycles can arise from different states in BRN and move towards deadlock state which leads the system to BC metastasis.

In homeostatic condition, the level of PTEN and p53 are expressed as active in state (0,0,0,1,0,1) while the other genes such as CXCL12, CXCR4, PDZK1 and PI3k/Akt remain in the oscillations. While the last and most critical states (1, 1, 1, 0, 0, 1), (1, 1, 0, 0, 1, 1), (1, 1, 0, 0, 1, 0) and (1, 1,1, 0, 1, 0) represent higher levels of CXCL12 and CXCR4, where the system move towards deadlock state (1, 1, 1, 0, 1, 0) (Fig. 6). The increased expression of CXCL12 in signal transduction pathway play a significant role in cancer progression through PI3k and Akt signaling (Teicher & Fricker, 2010). It is identified from the state graph that the interaction of ligand CXCL12 with receptor CXCR4 is initiated following the activation of PDZK1, PI3k and Akt that finally cause down-regulation of TSGs such as PTEN and p53. It was concluded that the mechanism of CXCL12 associated BRN is controlled by the inhibition of complex targets such as CXCL12/CXCR4, PDZK1 and PI3k/Akt to obtain new biological insight to treat metastatic BC.

## Discussion

In Pakistan, BC is the most frequently diagnosed cancer among females (Gilani, Kamal & Akhter, 2003; Menhas & Umer, 2015). It is caused by alteration of genes or proteins due to various environmental and genetic factors. De-regulation of these genes or proteins causes disruption of complex biological processes leading the system towards tumorigenesis and ultimately metastasis. CXCL12 plays a pivotal in different stages of tumor development and cancer metastasis (Cavallaro, 2013; Chang et al., 2009; Kobayashi et al., 2010). Interestingly, previous studies have demonstrated that, unlike the Caucasian population, the SNP rs1801157 of CXCL12 gene increases cancer susceptibility among Asians (Jin et al., 2012; Yaal-Hahoshen et al., 2006). Furthermore, this SNP is implicated in various cancers, human immunodeficiency virus (HIV) infection and type 1 diabetes (Arenzana-Seisdedos, 2015; Chen et al., 2006; Clegg et al., 2000; Hidalgo-Pascual et al., 2007; Luker & Luker, 2006; Maley et al., 2009; Pemberton et al., 2006; Qin et al., 2017; Sun et al., 2010; Sun et al., 2014). Therefore, in this study, prevalence of SNP rs1801157 of CXCL12 gene was investigated among 218 patients of BC and healthy individuals (*N* = 147) of Pakistani origin. The analysis of allelic frequencies of distribution showed a significant difference among the patients of BC and non-BC individuals (*p* < 0.05) as shown in Table 1 and Fig. 3. Although the genotypic frequencies indicated that the prevalence of genotype GG (*p* < 0.01) among patients of BC was more significant as compared to genotype AA (*n* = 21) (Table 2 and Fig. 3). Our findings suggest that allele G is possibly increasing the risk of BC. Unlike most of the studies has shown allele A to be a BC risk factor (De Oliveira et al., 2009; Sei et al., 2001; Voevodin, Samilchuk & Dashti, 1999; Wang et al., 2011; Winkler et al., 1998). It has investigated that genetic polymorphisms of CXCL12 involving various environmental factors in the growth and development of tumor pathogenesis.

Chemokines are known to induce inflammation and play a role in tumor growth by changing the tumor micro-environment and metastasis to other organs such as liver, lung, brain or bone (Cavallaro, 2013; Liberman et al., 2012; Thelen & Stein, 2008; Wang, Loberg & Taichman, 2006). Previous studies have analyzed that higher expression level of *CXCL12* lead the breast tumor towards metastasis (Jin et al., 2012; Kobayashi et al., 2010). Few studies showed that SNP rs1801157 of CXCL12 gene may be a prognostic marker of BC lymphocytic metastatic cells (Chang et al., 2009; Sun et al., 2014). This study was conducted in Pakistani population to find the no significance association of genotype GG with various BC stages (I, II, III and IV) (*p* = 0.369) (Table 3, Fig. 4A) and organ (lung, liver, bones and brain) metastasis (*p* = 0.349) (Table 3, Fig. 4B). Previous meta-analysis of genome-wide studies have indicated that the relationship between SNP rs1801157 of CXCL12 gene and cancer risk have some limitations due to their limited number of subjects and absence of different types of population in one study. Our MLR analysis demonstrated that weight of an individuals, affected lymph nodes, hormonal status (estrogen and progesterone receptor) and family history associated with the GG genotype (GG:AA, odds ratio (OR) = 1.30, 95% CI [1.06–1.60]) (shown in Table 4) were independent risk factors in the pathophysiology of BC. Our study provides a new biological insight into the prevalence of SNP rs1801157 of CXCL12 gene in Pakistani population and its association with possible gene-gene and gene-environment interaction.

A SNP (rs1801157; G/A) has essential biological role in association with CXCL12 gene transcription (De Oliveira et al., 2009). In one of studies on prostate cancer patients, it was concluded that combined effect of SNP rs1801157 of CXCL12 gene associated with p53 codon lead the system towards cancer progression via a tumor micro-environment (Hirata et al., 2007). Chemokines play an important role in facilitating intracellular signaling after binding with CXCR4 to promote cell proliferation (Begley et al., 2015; Printz, 2012; Sun et al., 2014). Increased expression of CXCL12–CXCR4 has been identified in a number of diseases such as systemic lupus erythematous (SLE), ankylosing spondylitis (AS), osteoarthritis, mycosis fungoid (MF) and Inflammatory bowel disease (IBD) (Maj et al., 2015; Schimanski et al., 2011; Xu et al., 2007). In accordance with this study, the expression level of complex CXCL12–CXCR4 signaling was interlinked with regulation of proteins such as PDZK1, PTEN, PI3k/Akt and p53 which are involved in breast tumor metastasis (Fig. 6). These proteins have a set logical parameters to generate a state graph by *in-silico* analysis of CXCL12 associated BRN model (Fig. 5). It shows that the level of CXCL12, CXCR4, PDZK1 and PI3k/Akt are high that cause loss of function mutation of PTEN and p53 proteins involved in increased risk of BC growth and development. The increased expression of CXCL12–CXCR4 signaling activates PI3k/Akt, PDZK1 proteins, which cross-linked with insulin and estrogen dependent signaling pathways such as IGF-1R and EGFR. Previously it has been reported that up-regulated expression of AKT and PI3k which in turn activates hormonal receptor ER-α, resulting in increased expression of IGF-1R and EGFR (Law et al., 2008; Pollak, 2008). These findings in our research emphasize the prognostic importance of SNP rs1801157 of CXCL12 gene in Pakistani population and association of CXCL12 with complex dynamical interactions among genes/proteins involved in cell cycle regulation. Therefore it can be concluded that CXCL12–CXCR4, PDZK1 and PI3k/Akt could serve as important therapeutic strategies for the treatment of BC.

## Conclusion

This study is reporting for the first time on CXCL12 rs1801157 single nucleotide polymorphism (SNP) in breast cancer (BC) patients in the Pakistani population. Our study also revealed the prognostic importance of rs1801157 polymorphism in BC patients of Pakistan. According to our findings, the genotype GG is more prevalent in BC patients as compared to AA while genotype AG has been found to be more prevalent in the non-BC. Keeping in view these two results, we can suggest that allele G in our population is possibly increasing the risk of BC. Together with the previous studies and our observations, the rs1801157 SNP of CXCL12 gene will help devise better prognostic strategies for BC. Previously it has observed that the chemotactic gradient CXCL12–CXCR4 axis plays a role in tumor growth by changing the tumor micro-environment that cause metastasis to the site of inflammation. Due to a known metastatic function of chemokine, SNP rs1801157 of CXCL12 gene has been found to be associated with higher risk of BC (Chang et al., 2009; Sun et al., 2014). Our multivariable logistic regression analysis shows a number of variables including age, weight, affected lymph nodes, hormonal status (estrogen and progesterone receptor) and family history associated with the GG genotype (GG:AA, odds ratio (OR) = 1.30, 95% CI [1.06–1.60]) was found to be independent risk factors for BC. Our *in-silico* study provides a new biological insight to analyze the expression level of complex CXCL12–CXCR4 signaling which was interlinked with regulation of proteins such as PDZK1, PTEN, PI3k/Akt and p53 involved in breast tumor metastasis. Therefore it is concluded that CXCL12 plays an important role in facilitating intracellular signaling after binding with CXCR4 to promote cell proliferation by using the Bioinformatic tools. We also suggest that CXCL12–CXCR4, PDZK1 and PI3k/Akt can serve as important therapeutic strategies for the treatment of BC.

## Supplemental Information

10.7717/peerj.3822/supp-1 Table S1 The state graph containing 64 states and 51 cyclic trajectories (representing as directed arrows among states)

## Additional Information and Declarations

### Data Availability

The following information was supplied regarding data availability: The raw data has been provided as a Supplemental File.
