# Supplementary material for: Association of rs1801157 single nucleotide polymorphism of CXCL12 gene in breast cancer in Pakistan and in-silico expression analysis of CXCL12–CXCR4 associated biological regulatory network
Source: PeerJ. 2017 Sep 15;5:e3822. doi: 10.7717/peerj.3822 (PMC5602684; doi:10.7717/peerj.3822)
Supplement: Table S1 [file peerj-05-3822-s001.pdf]

Table S1. It shows the state graph contained 64 states and 51 cyclic trajectories (representing as directed arrows among states).

Cycle No.	States
**0**	[[0, 0, 0, 0, 0, 0], [0, 0, 0, 1, 0, 0], [0, 0, 0, 1, 0, 1], [0, 0, 0, 0, 0, 1], [0, 0, 0, 0, 0, 0]]
1	[[0, 0, 0, 0, 1, 0], [0, 0, 0, 1, 1, 0], [0, 0, 0, 1, 1, 1], [0, 0, 0, 0, 1, 1], [0, 0, 0, 0, 1, 0]]
2	[[0, 0, 1, 0, 0, 0], [0, 0, 1, 1, 0, 0], [0, 0, 1, 1, 0, 1], [0, 0, 1, 0, 0, 1], [0, 0, 1, 0, 0, 0]]
3	[[0, 1, 0, 0, 0, 0], [0, 1, 1, 0, 0, 0], [0, 1, 1, 1, 0, 0], [0, 1, 0, 1, 0, 0], [0, 1, 0, 1, 0, 1], [0, 1, 0, 0, 0, 1], [0, 1, 0, 0, 0, 0]]
4	[[0, 1, 0, 0, 0, 0], [0, 1, 1, 0, 0, 0], [0, 1, 1, 1, 0, 0], [0, 1, 1, 1, 0, 1], [0, 1, 0, 1, 0, 1], [0, 1, 0, 0, 0, 1], [0, 1, 0, 0, 0, 0]]
5	[[0, 1, 0, 0, 0, 0], [0, 1, 0, 1, 0, 0], [0, 1, 0, 1, 0, 1], [0, 1, 0, 0, 0, 1], [0, 1, 0, 0, 0, 0]]
6	[[0, 1, 0, 0, 0, 1], [0, 1, 1, 0, 0, 1], [0, 1, 1, 0, 0, 0], [0, 1, 1, 1, 0, 0], [0, 1, 0, 1, 0, 0], [0, 1, 0, 1, 0, 1], [0, 1, 0, 0, 0, 1]]
7	[[0, 1, 0, 0, 0, 1], [0, 1, 1, 0, 0, 1], [0, 1, 1, 0, 0, 0], [0, 1, 1, 1, 0, 0], [0, 1, 1, 1, 1, 0], [0, 1, 0, 1, 1, 0], [0, 1, 0, 1, 0, 0], [0, 1, 0, 1, 0, 1], [0, 1, 0, 0, 0, 1]]
8	[[0, 1, 0, 0, 0, 1], [0, 1, 1, 0, 0, 1], [0, 1, 1, 0, 0, 0], [0, 1, 1, 1, 0, 0], [0, 1, 1, 1, 1, 0], [0, 1, 0, 1, 1, 0], [0, 1, 0, 1, 1, 1], [0, 1, 0, 1, 0, 1], [0, 1, 0, 0, 0, 1]]
9	[[0, 1, 0, 0, 0, 1], [0, 1, 1, 0, 0, 1], [0, 1, 1, 0, 0, 0], [0, 1, 1, 1, 0, 0], [0, 1, 1, 1, 1, 0], [0, 1, 1, 1, 1, 1], [0, 1, 0, 1, 1, 1], [0, 1, 0, 1, 0, 1], [0, 1, 0, 0, 0, 1]]
10	[[0, 1, 0, 0, 0, 1], [0, 1, 1, 0, 0, 1], [0, 1, 1, 0, 0, 0], [0, 1, 1, 1, 0, 0], [0, 1, 1, 1, 0, 1], [0, 1, 0, 1, 0, 1], [0, 1, 0, 0, 0, 1]]
11	[[0, 1, 0, 0, 0, 1], [0, 1, 1, 0, 0, 1], [0, 1, 1, 0, 0, 0], [0, 1, 1, 1, 0, 0], [0, 1, 1, 1, 0, 1], [0, 1, 1, 1, 1, 1], [0, 1, 0, 1, 1, 1], [0, 1, 0, 1, 0, 1], [0, 1, 0, 0, 0, 1]]

12	[[0, 1, 0, 0, 0, 1], [0, 1, 0, 0, 0, 0], [0, 1, 1, 0, 0, 0], [0, 1, 1, 1, 0, 0], [0, 1, 1, 1, 1, 0], [0, 1, 0, 1, 1, 0], [0, 1, 0, 1, 0, 0], [0, 1, 0, 1, 0, 1], [0, 1, 0, 0, 0, 1]]
13	[[0, 1, 0, 0, 0, 1], [0, 1, 0, 0, 0, 0], [0, 1, 1, 0, 0, 0], [0, 1, 1, 1, 0, 0], [0, 1, 1, 1, 1, 0], [0, 1, 0, 1, 1, 0], [0, 1, 0, 1, 1, 1], [0, 1, 0, 1, 0, 1], [0, 1, 0, 0, 0, 1]]
14	[[0, 1, 0, 0, 0, 1], [0, 1, 0, 0, 0, 0], [0, 1, 1, 0, 0, 0], [0, 1, 1, 1, 0, 0], [0, 1, 1, 1, 1, 0], [0, 1, 1, 1, 1, 1], [0, 1, 0, 1, 1, 1], [0, 1, 0, 1, 0, 1], [0, 1, 0, 0, 0, 1]]
15	[[0, 1, 0, 0, 0, 1], [0, 1, 0, 0, 0, 0], [0, 1, 1, 0, 0, 0], [0, 1, 1, 1, 0, 0], [0, 1, 1, 1, 0, 1], [0, 1, 1, 1, 1, 1], [0, 1, 0, 1, 1, 1], [0, 1, 0, 1, 0, 1], [0, 1, 0, 0, 0, 1]]
16	[[0, 1, 0, 0, 1, 0], [0, 1, 0, 1, 1, 0], [0, 1, 0, 1, 0, 0], [0, 1, 0, 1, 0, 1], [0, 1, 0, 0, 0, 1], [0, 1, 1, 0, 0, 1], [0, 1, 1, 0, 0, 0], [0, 1, 1, 1, 0, 0], [0, 1, 1, 1, 1, 0], [0, 1, 1, 1, 1, 1], [0, 1, 0, 1, 1, 1], [0, 1, 0, 0, 1, 1], [0, 1, 0, 0, 1, 0]]
17	[[0, 1, 0, 0, 1, 0], [0, 1, 0, 1, 1, 0], [0, 1, 0, 1, 0, 0], [0, 1, 0, 1, 0, 1], [0, 1, 0, 0, 0, 1], [0, 1, 1, 0, 0, 1], [0, 1, 1, 0, 0, 0], [0, 1, 1, 1, 0, 0], [0, 1, 1, 1, 0, 1], [0, 1, 1, 1, 1, 1], [0, 1, 0, 1, 1, 1], [0, 1, 0, 0, 1, 1], [0, 1, 0, 0, 1, 0]]
18	[[0, 1, 0, 0, 1, 0], [0, 1, 0, 1, 1, 0], [0, 1, 0, 1, 0, 0], [0, 1, 0, 1, 0, 1], [0, 1, 0, 0, 0, 1], [0, 1, 0, 0, 1, 1], [0, 1, 0, 0, 1, 0]]
19	[[0, 1, 0, 0, 1, 0], [0, 1, 0, 1, 1, 0], [0, 1, 0, 1, 0, 0], [0, 1, 0, 1, 0, 1], [0, 1, 0, 0, 0, 1], [0, 1, 0, 0, 0, 0], [0, 1, 1, 0, 0, 0], [0, 1, 1, 1, 0, 0], [0, 1, 1, 1, 1, 0], [0, 1, 1, 1, 1, 1], [0, 1, 0, 1, 1, 1], [0, 1, 0, 0, 1, 1], [0, 1, 0, 0, 1, 0]]
20	[[0, 1, 0, 0, 1, 0], [0, 1, 0, 1, 1, 0], [0, 1, 0, 1, 0, 0], [0, 1, 0, 1, 0, 1], [0, 1, 0, 0, 0, 1], [0, 1, 0, 0, 0, 0], [0, 1, 1, 0, 0, 0], [0, 1, 1, 1, 0, 0], [0, 1, 1, 1, 0, 1], [0, 1, 1, 1, 1, 1], [0, 1, 0, 1, 1, 1], [0, 1, 0, 0, 1, 1], [0, 1, 0, 0, 1, 0]]
21	[[0, 1, 0, 0, 1, 0], [0, 1, 0, 1, 1, 0], [0, 1, 0, 1, 0, 0], [0, 1, 0, 1, 0, 1], [0, 1, 0, 0, 0, 1], [0, 1, 0, 0, 0, 0], [0, 1, 0, 0, 1, 0]]
22	[[0, 1, 0, 0, 1, 0], [0, 1, 0, 1, 1, 0], [0, 1, 0, 1, 1, 1], [0, 1, 0, 0, 1, 1], [0, 1, 0, 0, 1, 0]]

23	[[0, 1, 0, 0, 1, 0], [0, 1, 0, 1, 1, 0], [0, 1, 0, 1, 1, 1], [0, 1, 0, 1, 0, 1], [0, 1, 0, 0, 0, 1], [0, 1, 0, 0, 1, 1], [0, 1, 0, 0, 1, 0]]
24	[[0, 1, 0, 0, 1, 0], [0, 1, 0, 1, 1, 0], [0, 1, 0, 1, 1, 1], [0, 1, 0, 1, 0, 1], [0, 1, 0, 0, 0, 1], [0, 1, 0, 0, 0, 0], [0, 1, 0, 0, 1, 0]]
25	[[0, 1, 1, 0, 0, 0], [0, 1, 1, 1, 0, 0], [0, 1, 1, 1, 0, 1], [0, 1, 1, 0, 0, 1], [0, 1, 1, 0, 0, 0]]
26	[[1, 0, 0, 0, 0, 0], [1, 0, 0, 1, 0, 0], [1, 0, 0, 1, 0, 1], [1, 0, 0, 0, 0, 1], [1, 0, 0, 0, 0, 0]]
27	[[1, 0, 0, 0, 1, 0], [1, 0, 0, 1, 1, 0], [1, 0, 0, 1, 1, 1], [1, 0, 0, 0, 1, 1], [1, 0, 0, 0, 1, 0]]
28	[[1, 0, 1, 0, 0, 0], [1, 0, 1, 1, 0, 0], [1, 0, 1, 1, 0, 1], [1, 0, 1, 0, 0, 1], [1, 0, 1, 0, 0, 0]]
29	[[1, 1, 0, 0, 0, 0], [1, 1, 1, 0, 0, 0], [1, 1, 1, 1, 0, 0], [1, 1, 0, 1, 0, 0], [1, 1, 0, 1, 0, 1], [1, 1, 0, 0, 0, 1], [1, 1, 0, 0, 0, 0]]
30	[[1, 1, 0, 0, 0, 0], [1, 1, 1, 0, 0, 0], [1, 1, 1, 1, 0, 0], [1, 1, 1, 1, 0, 1], [1, 1, 0, 1, 0, 1], [1, 1, 0, 0, 0, 1], [1, 1, 0, 0, 0, 0]]
31	[[1, 1, 0, 0, 0, 0], [1, 1, 0, 1, 0, 0], [1, 1, 0, 1, 0, 1], [1, 1, 0, 0, 0, 1], [1, 1, 0, 0, 0, 0]]
32	[[1, 1, 0, 0, 0, 1], [1, 1, 1, 0, 0, 1], [1, 1, 1, 0, 0, 0], [1, 1, 1, 1, 0, 0], [1, 1, 0, 1, 0, 0], [1, 1, 0, 1, 0, 1], [1, 1, 0, 0, 0, 1]]
33	[[1, 1, 0, 0, 0, 1], [1, 1, 1, 0, 0, 1], [1, 1, 1, 0, 0, 0], [1, 1, 1, 1, 0, 0], [1, 1, 1, 1, 1, 0], [1, 1, 0, 1, 1, 0], [1, 1, 0, 1, 0, 0], [1, 1, 0, 1, 0, 1], [1, 1, 0, 0, 0, 1]]
34	[[1, 1, 0, 0, 0, 1], [1, 1, 1, 0, 0, 1], [1, 1, 1, 0, 0, 0], [1, 1, 1, 1, 0, 0], [1, 1, 1, 1, 1, 0], [1, 1, 0, 1, 1, 0], [1, 1, 0, 1, 1, 1], [1, 1, 0, 1, 0, 1], [1, 1, 0, 0, 0, 1]]
35	[[1, 1, 0, 0, 0, 1], [1, 1, 1, 0, 0, 1], [1, 1, 1, 0, 0, 0], [1, 1, 1, 1, 0, 0], [1, 1, 1, 1, 1, 0], [1, 1, 1, 1, 1, 1], [1, 1, 0, 1, 1, 1], [1, 1, 0, 1, 0, 1], [1, 1, 0, 0, 0, 1]]
36	[[1, 1, 0, 0, 0, 1], [1, 1, 1, 0, 0, 1], [1, 1, 1, 0, 0, 0], [1, 1, 1, 1, 0, 0], [1, 1, 1, 1, 0, 1], [1, 1, 0, 1, 0, 1], [1, 1, 0, 0, 0, 1]]
37	[[1, 1, 0, 0, 0, 1], [1, 1, 1, 0, 0, 1], [1, 1, 1, 0, 0, 0], [1, 1, 1, 1, 0, 0], [1, 1, 1, 1, 0, 1], [1, 1, 1, 1, 1,

	1], [1, 1, 0, 1, 1, 1], [1, 1, 0, 1, 0, 1], [1, 1, 0, 0, 0, 1]]
38	[[1, 1, 0, 0, 0, 1], [1, 1, 0, 0, 0, 0], [1, 1, 1, 0, 0, 0], [1, 1, 1, 1, 0, 0], [1, 1, 1, 1, 1, 0], [1, 1, 0, 1, 1, 0], [1, 1, 0, 1, 0, 0], [1, 1, 0, 1, 0, 1], [1, 1, 0, 0, 0, 1]]
39	[[1, 1, 0, 0, 0, 1], [1, 1, 0, 0, 0, 0], [1, 1, 1, 0, 0, 0], [1, 1, 1, 1, 0, 0], [1, 1, 1, 1, 1, 0], [1, 1, 0, 1, 1, 0], [1, 1, 0, 1, 1, 1], [1, 1, 0, 1, 0, 1], [1, 1, 0, 0, 0, 1]]
40	[[1, 1, 0, 0, 0, 1], [1, 1, 0, 0, 0, 0], [1, 1, 1, 0, 0, 0], [1, 1, 1, 1, 0, 0], [1, 1, 1, 1, 1, 0], [1, 1, 1, 1, 1, 1], [1, 1, 0, 1, 1, 1], [1, 1, 0, 1, 0, 1], [1, 1, 0, 0, 0, 1]]
41	[[1, 1, 0, 0, 0, 1], [1, 1, 0, 0, 0, 0], [1, 1, 1, 0, 0, 0], [1, 1, 1, 1, 0, 0], [1, 1, 1, 1, 0, 1], [1, 1, 1, 1, 1, 1], [1, 1, 0, 1, 1, 1], [1, 1, 0, 1, 0, 1], [1, 1, 0, 0, 0, 1]]
42	[[1, 1, 0, 0, 1, 0], [1, 1, 0, 1, 1, 0], [1, 1, 0, 1, 0, 0], [1, 1, 0, 1, 0, 1], [1, 1, 0, 0, 0, 1], [1, 1, 1, 0, 0, 1], [1, 1, 1, 0, 0, 0], [1, 1, 1, 1, 0, 0], [1, 1, 1, 1, 1, 0], [1, 1, 1, 1, 1, 1], [1, 1, 0, 1, 1, 1], [1, 1, 0, 0, 1, 1], [1, 1, 0, 0, 1, 0]]
43	[[1, 1, 0, 0, 1, 0], [1, 1, 0, 1, 1, 0], [1, 1, 0, 1, 0, 0], [1, 1, 0, 1, 0, 1], [1, 1, 0, 0, 0, 1], [1, 1, 1, 0, 0, 1], [1, 1, 1, 0, 0, 0], [1, 1, 1, 1, 0, 0], [1, 1, 1, 1, 0, 1], [1, 1, 1, 1, 1, 1], [1, 1, 0, 1, 1, 1], [1, 1, 0, 0, 1, 1], [1, 1, 0, 0, 1, 0]]
44	[[1, 1, 0, 0, 1, 0], [1, 1, 0, 1, 1, 0], [1, 1, 0, 1, 0, 0], [1, 1, 0, 1, 0, 1], [1, 1, 0, 0, 0, 1], [1, 1, 0, 0, 1, 1], [1, 1, 0, 0, 1, 0]]
45	[[1, 1, 0, 0, 1, 0], [1, 1, 0, 1, 1, 0], [1, 1, 0, 1, 0, 0], [1, 1, 0, 1, 0, 1], [1, 1, 0, 0, 0, 1], [1, 1, 0, 0, 0, 0], [1, 1, 1, 0, 0, 0], [1, 1, 1, 1, 0, 0], [1, 1, 1, 1, 1, 0], [1, 1, 1, 1, 1, 1], [1, 1, 0, 1, 1, 1], [1, 1, 0, 0, 1, 1], [1, 1, 0, 0, 1, 0]]
46	[[1, 1, 0, 0, 1, 0], [1, 1, 0, 1, 1, 0], [1, 1, 0, 1, 0, 0], [1, 1, 0, 1, 0, 1], [1, 1, 0, 0, 0, 1], [1, 1, 0, 0, 0, 0], [1, 1, 1, 0, 0, 0], [1, 1, 1, 1, 0, 0], [1, 1, 1, 1, 0, 1], [1, 1, 1, 1, 1, 1], [1, 1, 0, 1, 1, 1], [1, 1, 0, 0, 1, 1], [1, 1, 0, 0, 1, 0]]
47	[[1, 1, 0, 0, 1, 0], [1, 1, 0, 1, 1, 0], [1, 1, 0, 1, 0, 0], [1, 1, 0, 1, 0, 1], [1, 1, 0, 0, 0, 1], [1, 1, 0, 0, 0, 0], [1, 1, 0, 0, 1, 0]]

48	[[1, 1, 0, 0, 1, 0], [1, 1, 0, 1, 1, 0], [1, 1, 0, 1, 1, 1], [1, 1, 0, 0, 1, 1], [1, 1, 0, 0, 1, 0]]
49	[[1, 1, 0, 0, 1, 0], [1, 1, 0, 1, 1, 0], [1, 1, 0, 1, 1, 1], [1, 1, 0, 1, 0, 1], [1, 1, 0, 0, 0, 1], [1, 1, 0, 0, 1, 1], [1, 1, 0, 0, 1, 0]]
50	[[1, 1, 0, 0, 1, 0], [1, 1, 0, 1, 1, 0], [1, 1, 0, 1, 1, 1], [1, 1, 0, 1, 0, 1], [1, 1, 0, 0, 0, 1], [1, 1, 0, 0, 0, 0], [1, 1, 0, 0, 1, 0]]
51	[[1, 1, 1, 0, 0, 0], [1, 1, 1, 1, 0, 0], [1, 1, 1, 1, 0, 1], [1, 1, 1, 0, 0, 1], [1, 1, 1, 0, 0, 0]]
